# Construction of Metabolically Biotinylated Adenovirus with Deleted Fiber Knob as Targeting Vector

**DOI:** 10.1186/1743-422X-7-316

**Published:** 2010-11-12

**Authors:** Yanzheng Liu, Philippe Valadon, Jan E Schnitzer

**Affiliations:** 1 Proteogenomics Research Institute for Systems Medicine, 11107 Roselle St. San Diego, CA 92121 USA

## Abstract

Gene delivery vectors based on adenovirus, particularly human adenovirus serotype 5 (hAd5) have great potential for the treatment of variety of diseases. However, the tropism of hAd5 needs to be modified to achieve tissue- or cell- specific therapies for the successful application of this vector system to clinic. Here, we modified hAd5 tropism by replacing the fiber knob which contains the coxsackievirus B and adenovirus receptor (CAR)-binding sites with a biotin acceptor peptide, a truncated form of *Propionibacterium shermanii *1.3 S transcarboxylase domain (PSTCD), to enable metabolically biotinylation of the virus. We demonstrate here that the new adenovirus no longer shows CAR-dependent cell uptake and transduction. When metabolically biotinylated and avidin-coated, it forms a nano-complex that can be retargeted to distinct cells using biotinylated antibodies. This vector may prove useful in the path towards achieving targeted gene delivery.

## Findings

The hAd5-based vector remains one of the most popular vector systems for gene delivery and cancer gene therapy. However, the ubiquitous expression of adenoviral primary receptor CAR in many tissues and the predominant liver tropism of the vector after systemic administration limit the application of hAd5 vector to clinical use [1]. Therefore, strategies to re-direct hAd5 infection and to decrease the rapid uptake of the virus by the reticuloendothelial system (RES) will be essential for many gene therapy applications. The hAd5 binds to most cell types through the interaction of its fiber knob domain with cell surface CAR [2]. Retargeting of the hAd5 vector appears to be more effective when the transduction mediated by retargeting ligands is directed through the fiber protein [3]. The adenoviral capsid proteins, especially fiber knob and hexon, associate with blood factors and mediate hepatocyte transduction *in vivo *[4-8]. The binding of hAd5 hexon protein with coagulation factor FX plays a major role in hepatocyte infection *in vivo *[4-6]. Single point mutation within the hexon hypervariable regions effectively blocks FX-mediated adenoviral hepatocyte transduction *in vitro *and *in vivo *[7]. The hAd5 fiber knob domain binds coagulation factor IX and complement component C4-binding protein that bridge the virus to cognate receptors on hepatocytes [8]. In the same study, a modified adenoviral vector with fiber knob mutations was shown to have less accumulation in both hepatocyte and Kupffer cells. Therefore, both the fiber and hexon proteins need to be modified to retarget adenoviral vector away from the liver.

In the study reported here, we have ablated the native tropism of hAd5 by removing the fiber knob and part of the central shaft. We have added to the short fiber a truncated form of PSTCD as a biotin acceptor protein to allow the virus to be metabolically biotinylated. We demonstrated here that the N-terminal tail and 9 shaft repeats fused with PSTCD protein can be successfully incorporated into the adenovirus particles to form the required trimer and to be biotinylated. The resulting metabolically biotinylated adenovirus can be redirected to specific cells depending on the biotinylated antibody used.

**Figure 1:**
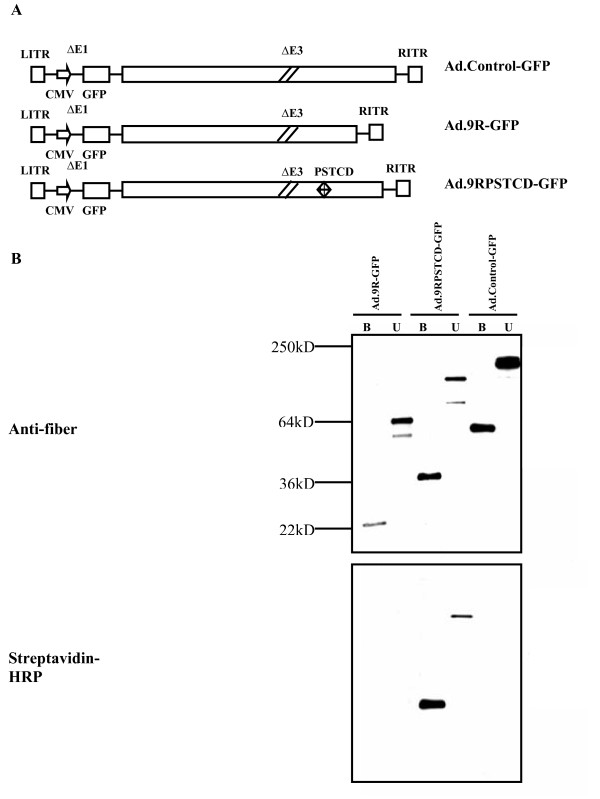
**Structure, fiber trimerization and biotinylation of new 9RPSTCD adenoviral vector**. **(A) **Structure of adenoviral vectors. All the viral vectors were replication-incompetent, E1,E3-deleted vector with a GFP as reporter gene, but each vector containing different fiber genes. The Ad.Control-GFP vector had a wild type fiber; the Ad.9R-GFP had a 9R modified fiber; the Ad.9RPSTCD-GFP had a 9R fused with PSTCD fiber. **(B) **Detection of biotinylation status of different viral vectors. Equal amount of purified viral vectors were loaded on SDS-PAGE gradient gels either boiled (B, to detect monomeric fibers) or unboiled (U, to detect trimeric fibers). The fiber protein and biotinylation were analyzed by western blotting using antibody against fiber protein or streptavidin-HRP. All of the viral fiber proteins formed trimers but only the virus with the PSTCD gene was biotinylated.

The hAd5 fiber proteins exist as homotrimers which contains an N-terminal tail, a central shaft comprising 21 repeating sequences of 15 amino acids, and a C-terminal globular knob domain [9]. Without the knob domain, a shortened fiber protein 9R containing the N-terminal tail and a shaft with 9 repeating sequences can form stable trimers and support peptide fusion [10]. Also a truncated form of PSTCD fused to the C-terminus of the fiber protein can be efficiently biotinylated by human holocarboxylase synthetase presented in HEK-293 cells [11]. Here, we fused the 70-amino-acid PSTCD on the 9R modified fiber. We found that the 9R modified fiber tolerates the large protein addition and remains fiber trimerization. The fused construction could readily be biotinylated in mammalian cells (data not shown). We then replaced the wild type fiber gene in hAd5 vector with the 9R or 9RPSTCD modified fiber gene to generate Ad.9R-GFP and Ad.9RPSTCD-GFP viral vectors using the Adeasy system [12] with modifications. Ad.Control-GFP which was E1/E3 deleted viral vector expressing GFP was constructed as previously described [13]. The structure organization of each vector is illustrated in Figure 1A.

The presence of the modified fiber and the successful biotinylation of the fiber were confirmed using immunoblotting analysis. As shown in Figure 1B, all 3 fiber proteins trimerized and assembled onto the hAd5 viral particles, but only the 9RPSTCD protein could be biotinylated. Thus, the 9RPSTCD modified fiber assembled onto hAd5 particles and was metabolically biotinylated during virus production.

**Figure 2:**
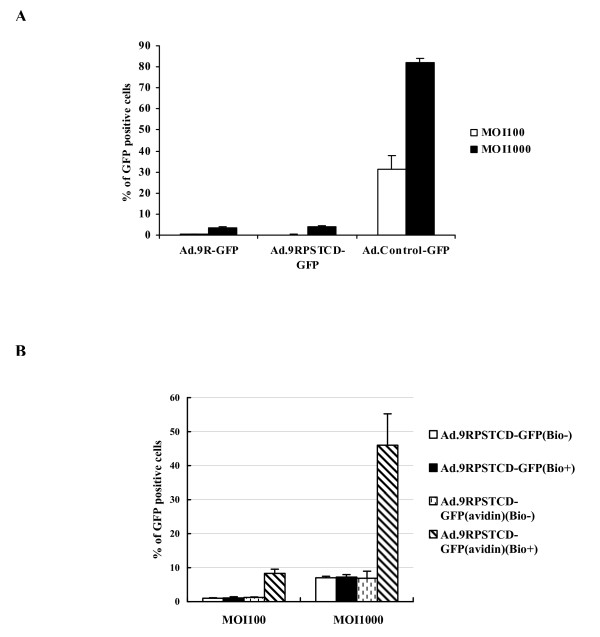
**Cell transduction was ablated in the Ad.9RPSTCD-GFP viral vector and was rescued through biotin-avidin-biotin interaction**. **(A, B) **The transduction efficiency of the indicated viral vectors to MA104/APP cells. Cells were collected and GFP-positive cells were counted by flow cytometry 24 h after transduction at different MOI (viral particles/cell). **(B) **The transduction efficiency of the Ad.9RPSTCD-GFP(avidin) vector to cells increased through the interaction of avidin on the Ad.9RPSTCD-GFP(avidin) vector with the biotin on the cell surface. Indicated viral vectors were added to chemically biotinylated MA104/APP cells (bio+) or unbiotinylated MA104/APP cells (bio-), and the GFP positive cells were counted 24 h later by flow cytometry. The data shown represent means ± SD of triplicate determinations of the GFP positive cells.

We then began to characterize our new viral vectors functionally. The interaction of the Ad5 fiber knob with cell surface CAR mainly determines the tropism of the virus in cell culture. Therefore, both the Ad.9R-GFP and Ad.9RPSTCD-GFP vectors should have decreased transduction efficiency to a cell line that has high expression level of CAR because the complete knob domain is deleted. We transduced MA104/APP cells [14] with indicated viral vectors at different multiplicity of infection (MOI). The cells were collected and GFP positive cells were counted by flow cytometry after 24 h. As shown in Figure 2A, unlike the unmodified control virus, both the Ad.9R-GFP and the Ad.9RPSTCD-GFP vectors failed to transduce MA104/APP cells. Thus, the normal tropism and transduction efficiency were successfully ablated in the new vectors.

To test the possibility that the Ad.9RPSTCD-GFP vector could be retargeted through biotin-avidin-biotin interaction in cells, we first pre-incubated the Ad9RPSTCD-GFP vector with neutravidin to generate the Ad.9RPSTCD-GFP(avidin) vector. This neutravidin-coated vector was purified by cesium chloride (CsCl) density gradient centrifugation. We then chemically biotinylated the surface proteins of MA104/APP cells and incubated them with the Ad.9RPSTCD-GFP(avidin) vector at different MOI before assessing transduction by counting GFP-positive cells. As shown in Figure 2B, the Ad.9RPSTCD-GFP(avidin) virus only transduced cells with biotinylated cell surface proteins (~ 50% cells expressed GFP at MOI 1000). If cells were not biotinylated, Ad.9RPSTCD-GFP(avidin) transduced less than 10% of the cells at the same MOI. The Ad.9RPSTCD-GFP virus could not, alone without the avidin coating, transduce native or surface-biotinylated cells, indicating that the biotin-avidin-biotin interaction is essential for efficient transduction.

**Figure 3:**
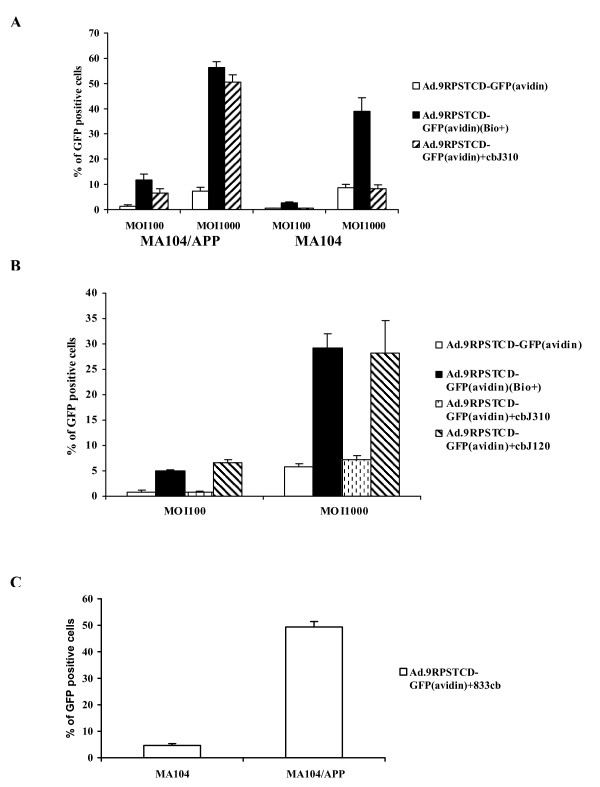
**Cell transduction by Ad.9RPSTCD-GFP(avidin) through biotinylated antibodies**. **(A) **The transduction efficiency of Ad.9RPSTCD-GFP(avidin) vector to MA104/APP or MA104 cells preincubated with biotinylated antibodies targeting rat APP. Cells were incubated with chemically biotinylated J310 antibodies against APP, then transduced with the viral vector. GFP positive cells were counted 24 h later. **(B) **The transduction efficiency of Ad.9RPSTCD-GFP(avidin) vector to RAEC cells preincubated with chemically biotinylated J310 antibodies against APP (non-specific) or J120 antibodies against CD34 (specific). **(C) **MA104/APP or MA104 cells transduced by Ad.9RPSTCD-GFP(avidin)-833cb complex.

Next, we tested whether the Ad.9RPSTCD-GFP(avidin) vector could be efficiently retargeted to cells by using biotinylated antibodies against a specific cell surface protein. We chemically biotinylated monoclonal antibody J310 which recognizes rat aminopeptidase P (APP) [15], and monoclonal antibody J120, which reacts specifically with CD34 [16]. Two cell lines, MA104 and its derivative cell line MA104/APP which stably expresses rat APP [14], were incubated first with biotinylated J310 and then with the Ad.9RPSTCD-GFP(avidin) vector. The transduced GFP positive cells were counted. Figure 3A shows that the Ad.9RPSTCD-GFP(avidin) transduced MA104/APP cells only when the cells were first incubated with the biotinylated J310 antibody. This vector did not effectively transduce MA104 cells not expressing APP even though they were still incubated with the biotinylated J310 antibody. Next, we performed same experiment using rat aortic endothelial cells (RAEC) that express abundant CD34 but not APP. The Ad.9RPSTCD-GFP(avidin) virus was able to successfully transduce RAEC when these cells were pre-incubated with chemically biotinylated J120 antibody but not with biotinylated J310 or no antibody at all (Figure 3B). For both experiments, we used surface-biotinylated cells as a positive control and noted that the transduction efficiency of specific antibodies-redirected vectors were very similar to it. These results further confirmed that viral targeting and transduction efficacy can be quite antibody-specific.

As a last test, we wanted to form preconjugated biotinylated adenovirus-avidin-biotinylated antibody nano-complexes in order to induce possible specific targeting. It is difficult to control the number and location of biotin residues when antibodies are chemically biotinylated. Without a defined biotin site, chemically biotinylated antibodies can be attached to avidin in any orientation, often interfering with their ability to bind antigens. In contrast, metabolically biotinylated Fab constructs can be genetically engineered to precisely control the number and location of biotin residues on the antibody. Our lab has developed metabolically biotinylated Fab fragments against rat APP (833cb Fab) which has one biotin residues located at the C-terminal side of the C1 domain of the heavy chain [17]. We incubated the Ad.9RPSTCD-GFP(avidin) viral vector with 833cb Fab and free Fab was removed by centrifugation-driven filtration using a 300K molecular weight cutoff filter. The final complexes, Ad.9RPSTCD-GFP(avidin)-833cb were used to transduce MA104/APP or MA104 cells, and the GFP positive cells were counted 24 h later. As shown in Figure 3C, Ad.9RPSTCD-GFP(avidin)-833cb transduced the MA104/APP cells but not MA104 cells under equivalent conditions. Thus, the transduction efficiency of the Ad.9RPSTCD-GFP(avidin) vector can be significantly increased through the binding to chemically biotinylated antibodies or biotinylated Fab against specific cell surface receptors.

Construction of targeted adenoviral vector through modification of fiber protein can be challenging because the trimerization of modified fiber is essential for proper virus assembly, and the retargeting ligands need to be functional in the trimerized fiber structure. In this report, we demonstrated that the 9RPSTCD modified fiber can be successfully assembled onto hAd5 virus and can be biotinylated simultaneously with viral production. Because of the deletion of CAR-interaction, the Ad.9RPSTCD-GFP(avidin) complex can be directed to transduce only selected cells by binding to other cell surface receptors through biotinylated targeting ligands. This 9RPSTCD virus may now be suitable for high throughput screening of targeting ligands *in vitro*. This vector may become useful for *in vivo *retargting and screening with future modifications that reduce RES uptake. For instance, we plan to further modify this vector by introducing the point mutation in the hexon protein to ablate the interaction of hexon with coagulation factor FX according the methods described in reference [7], and then evaluate RES uptake as well as possible retargeting *in vivo *with specific biotinylated antibodies. The methods and new vectors described and characterized here may form a foundation for future modifications and studies attempting to further improve tissue- and cell- specific retargeting of adenoviral vectors.

## List of abbreviations used

**hAd5**: human adenovirus serotype 5; **RES: **reticuloendothelial system; **CAR**: coxsackievirus B and adenovirus receptor; **PSTCD**: *Propionibacterium shermanii *1.3 S transcarboxylase domain; **MOI**: multiplicity of infection; **CsCl**: cesium chloride; **APP**: aminopeptidase P; **RAEC**: rat aortic endothelial cells.

## Competing interests

The authors declare that they have no competing interests.

## Authors' contributions

YZL conducted the major experiments related to this project and wrote the manuscript, PV provided the 833cb Fab, JES led the study and wrote the manuscript. All authors read and approved the final manuscript.
